# Mediator tail subunits can form amyloid-like aggregates *in vivo* and affect stress response in yeast

**DOI:** 10.1093/nar/gkv629

**Published:** 2015-07-02

**Authors:** Xuefeng Zhu, Lihua Chen, Jonas O. P. Carlsten, Qian Liu, Junsheng Yang, Beidong Liu, Claes M. Gustafsson

**Affiliations:** 1 Institute of Biomedicine, University of Gothenburg, P.O. Box 440, SE-405 30 Göteborg, Sweden; 2 Department of Chemistry and Molecular Biology, University of Gothenburg, Medicinaregatan 9C, SE-413 90 Göteborg, Sweden

## Abstract

The Med2, Med3 and Med15 proteins form a heterotrimeric subdomain in the budding yeast Mediator complex. This Med15 module is an important target for many gene specific transcription activators. A previous proteome wide screen in yeast identified Med3 as a protein with priogenic potential. In the present work, we have extended this observation and demonstrate that both Med3 and Med15 form amyloid-like protein aggregates under H_2_O_2_ stress conditions. Amyloid formation can also be stimulated by overexpression of Med3 or of a glutamine-rich domain present in Med15, which in turn leads to loss of the entire Med15 module from Mediator and a change in stress response. In combination with genome wide transcription analysis, our data demonstrate that amyloid formation can change the subunit composition of Mediator and thereby influence transcriptional output in budding yeast.

## INTRODUCTION

Amyloid-like aggregation of soluble proteins can result in cellular toxicity and cause neurodegenerative disorders (1). Amyloid formation may affect proteins involved in different biological functions, such as regulation of transcription and translation fidelity, as well as modulation of stress and drug resistance (2–6). The infectious form of prions can also induce amyloid formation and transform the normal protein into the misfolded, infectious version. Prions were first discovered in mammalian systems, but also exist in *Saccharomyces cerevisiae*, e.g. [URE3] and [PSI+], where they occur spontaneously at very low frequency. Yeast proteins with priogenic potential often contain domains rich in glutamine (Q) and/or asparagine (N). Overexpression of such domains or the entire prion protein can stimulate amyloid formation *in vivo* (7).

Mediator is a coregulator of eukaryotic transcription, which can function as a bridge between gene-specific transcription regulators and the RNA polymerase II (Pol II) transcription machinery at the promoter, but many other functions have also been ascribed to this multiprotein complex (8). Mediator is present in most eukaryotic organisms and its composition and structure have been studied in detail (9). One part of Mediator, denoted the ‘Med15 module’, is composed of Med2, Med3 (a.k.a. Pgd1) and Med15 (a.k.a. Gal11) (10–12). This triad of proteins is essential for the regulated transcription of many genes and it interacts with a number of transcription activators including Gal4, Gcn4, Pdr1, Oaf1 and others (13). Deletion of any one of the Med15 module subunits causes a destabilization of the module and an impaired transcription response to environmental changes (14,15). An interesting feature of the Med15 module proteins is the presence of Q and N-rich domains (16). Based on these sequence motifs, the Med15 module proteins were all analyzed in a previous screen for proteins with priogenic potential in the budding yeast proteome. Med3 was one of 24 proteins defined as priogenic, whereas Med2 and Med15 failed to pass the strict criteria used in this screen (17). The authors demonstrated that Med3 was able to form amyloids *in vivo*, which could replicate in cells as self-perpetuating epigenetic elements.

In the present work, we have extended the previous characterization of Med3 and also investigated how the Med15 module is affected by amyloid formation. We demonstrate Med3 and Med15, form amyloid-like protein aggregates under H_2_O_2_ stress condition. The C-terminal glutamine-rich domains (poly-Q) in both Med3 and Med15 are critical for this effect. Overexpression of full-length Med3 or the poly-Q domain of Med15 (Med15Q) promotes amyloid conversion of the endogenous proteins, which in turn leads to loss of the entire Med15 module. This dramatic effect on the Mediator structure changes the levels of many different transcripts and affects stress response. In combination with the previous report our data points to a protein-based mechanism that may regulate Mediator dependent transcriptional output (17).

## MATERIALS AND METHODS

### Yeast strains and growth conditions

Yeast strains used in this study are listed in Supplementary Table S1. Yeast cells were grown in yeast extract peptone dextrose (YPD) medium or synthetic drop-out media with supplements as indicated. Raffinose or galactose was used as carbon sources for overexpression experiments. For overexpression in yeast, DNA fragments encoding Med3, Med15Q and Med15C were amplified and cloned in frame to a N-terminal glutathione S-transferase tag (GST) in pEGH vector (18). DNA sequencing and immunoblotting was used to confirm the constructs. Plasmid transformation and overexpression were performed following standard protocols. Flag-Med18 construction was performed using polymerase chain reaction (PCR) amplification of the coding sequence of the C-terminus of the Med18(Srb5)-Flag tagged gene and the KanMX marker as previous described (19).

### *In* *situ* chromatin binding assay

Cells were grown to mid exponential phase, washed and resuspended in Buffer P (10 mM NaH_2_PO_4_ and 150 mM NaCl [pH 7.2]). Cells were incubated at room temperature for 30 min with or without Concanavalin A (Invitrogen, 1 mg/ml in 0.1 M NaHCO_3_ [pH 8.3]) at a final concentration of 0.1–0.2 mg/ml. After washing with Buffer P, cells were resuspended in EMMSorb buffer (15 mM KH phallate, 15 mM Na_2_HPO_4_, 90 mM NH_4_Cl, 1.2 M sorbitol [pH 7.0], 10 mM DTT and 0.125 mg/ml Zymolyase 100T) and incubated at room temperature for 10–20 min. Next, cells were washed twice in EMMsorb without zymolyase and once with lysis buffer (0.4 M Sorbitol, 150 mM NaAc, 2 mM MgAc_2_, 20 mM Pipes-KOH [pH 6.8], 1 × protease inhibitors (Thermo scientific, 78 847)). Cells stained with conA, were resuspended in lysis buffer at less than 8 × 10^8^ cells per ml and lysed by addition of Triton X-100 (final conc. 1%) and incubated for 5 min. The chromatin-enriched fraction was isolated by centrifugation (3000 rpm, Eppendorf 5424R, 5 min, room temperature), and the supernatant was carefully removed. Cells treated with conA and Triton X-100 were mixed in equal proportions with untreated cells. Mixed cells were stained with DAPI to visualize nuclear DNA (Region of Interest; ROI) and observed under widefield fluorescence microscopy (Carl Zeiss Axio Observer). The intensity of green fluorescent protein (GFP) colocalized with nuclear DNA was quantified in ConA stained and non-stained cells, respectively, using Zen (blue edition) software pixel density measurement. The ratio of the GFP to DAPI signal in conA stained cell was normalized to the same ratio in cells without conA staining. At least 50 cells with both GFP and DAPI signals were analyzed.

### Filter retardation assay

After 48 h induction, yeast cells were lysed using FastPrep as previously described (19). After incubation at 95°C for 5 min, both heated and non-heated lysates were incubated with 2% (w/v) sodium dodecyl sulphate (SDS) in 1 × phosphate buffered saline (PBS) for 10 min. All samples were applied to a 96-well dot blot system (Bio-Rad) with a cellulose acetate membrane (poor size 0.2 μm, GE healthcare) for detection of protein aggregates. In parallel, samples were applied to a nitrocellulose membrane for a total protein loading control. The membranes were washed five times with PBS with 0.1% SDS, followed by protein detection with immunoblotting using GST and GFP antibodies (27-4577-01, GE healthcare; AB290, Abcam).

### Thioflavin T staining

Amyloid staining with Thioflavin T (ThT) was performed following a published protocol (20), with minor changes. Cells were fixed in 50 mM KPO_4_ (pH 6.5), 1 mM MgCl_2_, 4% formaldehyde for 10 min and then washed three times with PM buffer (0.1 M KPO_4_ [pH 7.5], 1 mM MgCl_2_] and resuspended in PMST buffer (0.1 M KPO_4_ [pH 7.5], 1 mM MgCl_2_, 1 M Sorbitol, 0.1% Tween-20]. Next, cells were incubated with 0.125 mg/ml Zymolase at room temperature for 15 min in the presence of 0.6% β-mercaptoethanol. Spheroblasted cells were washed once and then resuspended in PMST and stained with 0.001% ThT for 20 min at room temperature. After washing five times with PMST, the cells were observed with a Zeiss Observer Z1 microscope using the cyan fluorescent protein (CFP) channel. At least 100 cells with simultaneously visible and the ThT and fluorescent signals (due to Med3-GFP, Med15-GFP or Htt103Q-mRFP) were analyzed. Cells with overlapping ThT and fluorescent signals were used as a marker for amyloid formation.

### Protein purification

Mediator was purified using a Flag-tag on Med18 from cells overexpressing GST, Med3 or Med15Q. The procedures for Mediator purification and analysis were as previously described (19). After centrifugation, the pellets were washed five times with wash buffer (20 mm Hepes-KOH, pH 7.6, 0.01% Nonidet P-40, 10% glycerol, 300 mm KOAc, 1 mm dithiothreitol and protease inhibitors) and detected by immunoblotting. The supernatants were used for assessing the composition of Mediator.

### RT-PCR and microarray expression analysis

RNA was prepared from yeast cells overexpressing GST or Med3-GST as previously described (21). RNA was further purified using the RNeasy purification kit (Qiagen). Quantitative RT-PCR were performed as previously described (22). Processing and hybridization using Affymetrix Yeast Genome 2.0 Arrays (Affymetrix, Santa Clara, CA, USA) were performed as per the manufacturer's instructions and three biological replicates were performed. Changes in gene expression were calculated by averaging log2 expression changes. False discovery rates were calculated using the ArrayStar (DNASTAR) software, after normalization within each experiment. Scatter plot were done in Prism. Gene ontology analysis of significantly affected genes was carried out using a web-based cluster interpreter program (Funspec, http://funspec.med.utoronto.ca/) and *P*-values < 0.001 were corrected for multiple category testing. Input data have been deposited in GEO as GSE65666.

### Growth curve measurements

Growth curves were measured by microcultivation experiments in triplicate at 30°C using the Bioscreen C system (Labsystems Oy, Helsinki, Finland). Overnight yeast cultures were inoculated in 350 μl ScGal-Ura with addition of sorbitol (1.5 M), NaCl (0.5 M) or Rapamycin (10 μM). The optical density was measured every 30 min for 72 h. Raw data in triplicate were used to get average values for plotting in Eexcel.

## RESULTS

### Mediator subunits form aggregates under H_2_O_2_ condition

**Figure 1.**
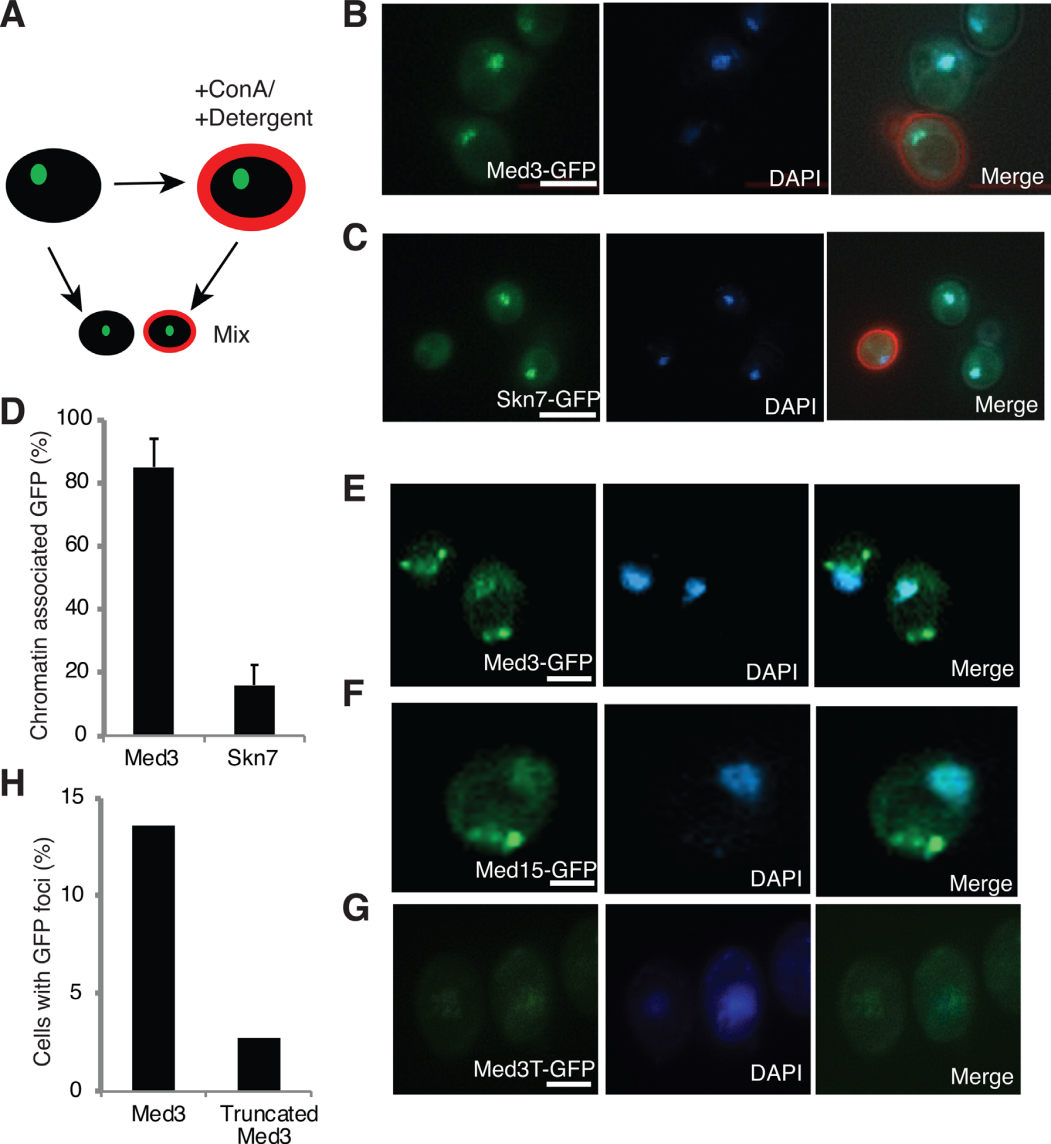
Mediator Med15 module subunits form aggregates under H_2_O_2_ stress condition. (**A**) Schematic outline of the experimental design for the *in situ* chromatin association assay. Cells expressing Med3-GFP or Skn7-GFP were stained with conA and subjected to detergent extraction. The treated cells were mixed with untreated cells, which served as total fluorescent signal control and observed using fluorescent microscopy. (**B** and **C**) Representative images of Med3-GFP and Skn7-GFP cells, stained with conA, showing association with chromatin. Scale bars = 1 μm. (**D**) Quantification of chromatin associated GFP fluorescent signals in Med3-GFP and Skn7-GFP cells treated with detergent. (**E** and **F**) Representative images of Med3-GFP and Med15-GFP expressing cells after exposure to H_2_O_2_ (0.03%) for 3 h, showing that GFP fluorescent foci do not overlap with nuclear DNA. (**G**) Truncated Med3-GFP (Med3T-GFP), lacking the poly-Q region does not form the fluorescent foci after exposure to H_2_O_2_. (**H**) The relative levels of fluorescent foci in Med3-GFP and Med3T-GFP cells after exposure to H_2_O_2_.

Mediator is required for transcriptional response to different stress conditions (23) and the complex is recruited to the promoters of stress induced genes (14). In wild-type (wt) cells, Mediator is exclusively located to the nucleus, where it overlaps with chromatin (24) and we wondered if cellular stress could change the subcellullar localization of Mediator subunits. To this end, we first investigated if the bulk of Mediator was associated with chromatin under normal conditions, using an *in situ* chromatin association assay (25). Mediator subunits were fused to GFP and expressed from their native promoters. The fusion proteins were functional, in as much as the yeast strain behaved as a normal, wt control (data not shown). As expected, Mediator subunits overlapped with DAPI staining of nuclear DNA (Supplementary Figure S1). In the chromatin association assay, detergent treatment (Triton X-100) was used to erase unstable chromatin interactions to investigate the stability of the Mediator-chromatin interaction (Figure 1A) (25). To ensure an unambiguous comparison of GFP fluorescence in detergent-extracted and non-extracted cells, concanavalin A (conA) staining were combined with the detergent-extract step. The red conA dye functioned as an indicator of detergent extracted cells. Non-extracted cells were mixed with equal numbers of extracted cells and observed in the same microscopic field (Figure 1A and B). For comparison, we analyzed the chromatin association of Skn7, a transcription activator, which is recruited to Tup1 associated promoters where Mediator also is found (26,27). The Mediator signal (Med3-GFP) remained nuclear and overlapped with DNA in the detergent-extracted cells stained with conA. In contrast, the chromatin association of Skn7-GFP was eliminated after detergent extraction (Figure 1C and D). Our data therefore demonstrate that Med3 and Mediator associate tightly with chromatin, under conditions where a DNA-binding transcription activator (Skn7) is lost from chromatin (Figure 1B).

We next analyzed if the nuclear localization of Med3-GFP was affected by different stress conditions (Supplementary Table S2) (28). In most cases, we could not observe a change in Mediator localization, but exposure to H_2_O_2_ led to the formation of cytoplasmic fluorescent Med3-GFP foci in a portion of the cells (14.2%) and the signal formed no longer overlapped with chromatin (Figure 1E). To follow up this observation, we analyzed the effects of H_2_O_2_ on a number of other Mediator subunits (Supplementary Figure S1). Interestingly, Med15-GFP, but no other Mediator subunits tested, also formed similar protein foci under H_2_O_2_ stress condition (Figure 1F and Supplementary Figure S1). Med3 and Med15 are closely associated in a subdomain of the Mediator complex. Both proteins contain glutamine (Q)-rich regions, but whereas Med15 contains multiple poly-Q domains spread over large sections of the protein, Med3 contains one single, well-defined C-terminal poly-Q domain (Supplementary Figure S2A and B). To verify the importance of this domain, we expressed a truncated version of Med3 fused to GFP. The truncated protein lacked the C-terminal 50 amino acids and was expressed to about half the levels of full-length Med3-GFP (Supplementary Figure S2C). In the truncated strain, we observed a significant decrease in the number of H_2_O_2_-induced fluorescent foci, suggesting that the poly-Q domain is important for foci formation. (Figure 1G and H).

### Overexpression of Med3 and Med15Q induce amyloid formation

**Figure 2.**
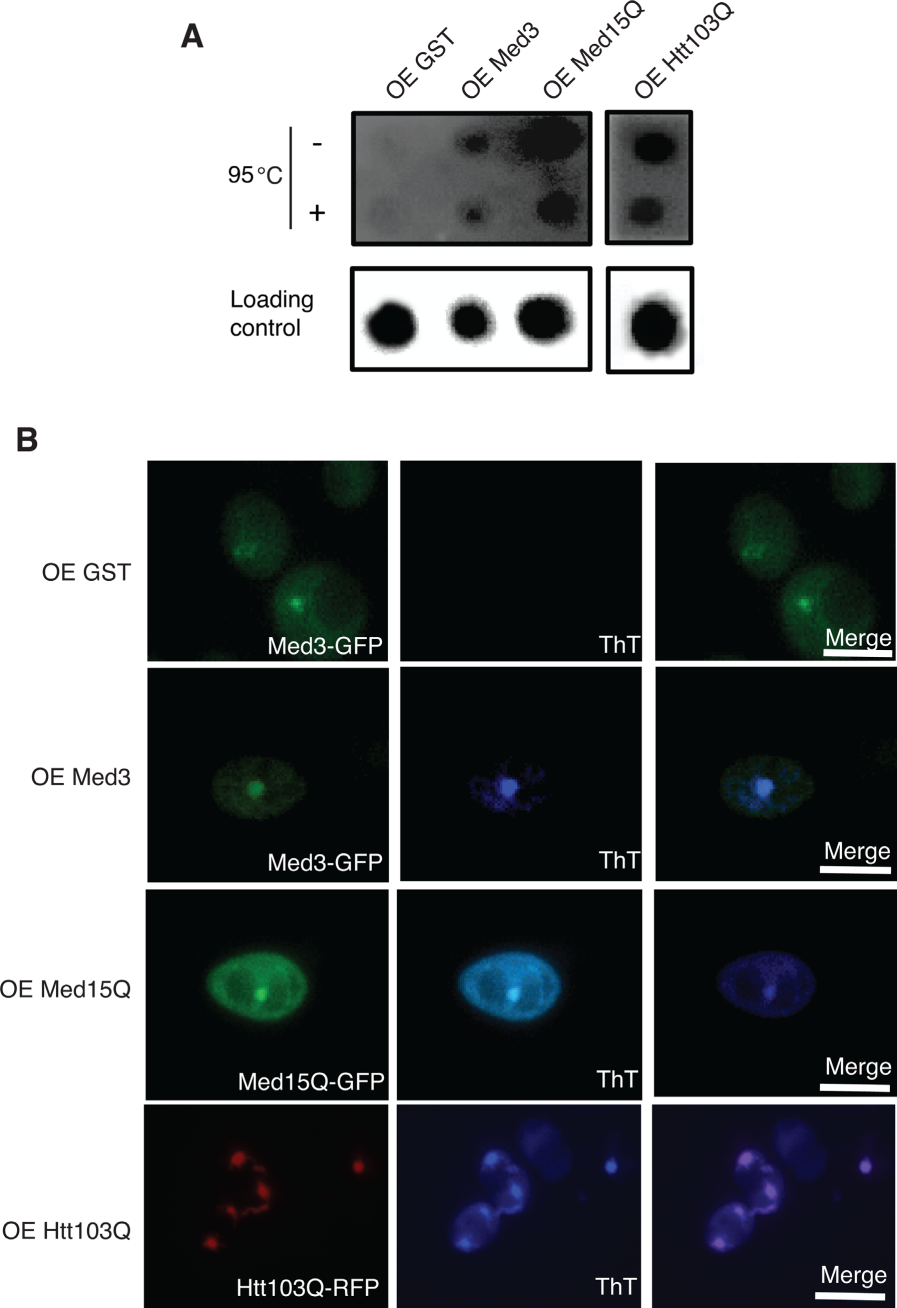
Overexpression of Med3 and Med15Q triggered amyloid-like aggregation. (**A**) Filter retardation assay showing aggregates in cell lysate after overexpression of Med3, Med15Q or Htt103Q. Equal amounts of cells lysates were loaded on cellulose acetate (upper panel) or nitrocellulose membranes (lower panel) as the loading control. In the upper panel, cell lysates are displayed with or without 95°C treatment for 5 min before incubation with SDS. (**B**) Thioflavin T (ThT) fluorescence staining showed amyloid-like aggregates induced by overexpression of Med3, Med15Q and Htt103Q, but not in a GST overexpression control.

H_2_O_2_ treatment induced protein foci, which could be explained by Med3 and Med15 amyloid aggregates. We next used plasmid-based overexpression of Mediator subunits to test whether overproduction induced protein aggregation (29). We overexpressed full-length Med3 to monitor amyloid formation *in vivo*. Med15 is a large protein and we did not manage to overexpress the full-length version, but instead expressed a 67 amino acids long poly-Q rich region of the protein (Med15Q, corresponding to amino acids 629 to 696 in full length Med15) (Supplementary Figure S2B and C). After 48 h induction, we analyzed amyloid formation using a filter retardation assay with the aggregation-prone Huntington's disease protein Htt103Q as a positive control. We found that overexpression of Med15Q led to the formation of prominent protein aggregates in the cells. Also Med3 overexpression caused protein aggregation, but the effect was more modest (Figure 2A). To demonstrate that the Med3/Med15Q aggregates result from amyloid-like aggregation, we used the amyloid specific dye ThT. As seen in Figure 2B, we found ThT signals colocalize with fluorescent foci. The formation of these aggregates was time dependent, since the ThT signal for Mediator proteins was observed only after 48 hrs of protein overexpression. Taken together, our results demonstrated that Med3 and Med15Q, when overproduced, form amyloid-like aggregates.

### Overexpressed Med3/Med15Q aggregates eliminate endogenous Mediator subunits

**Figure 3.**
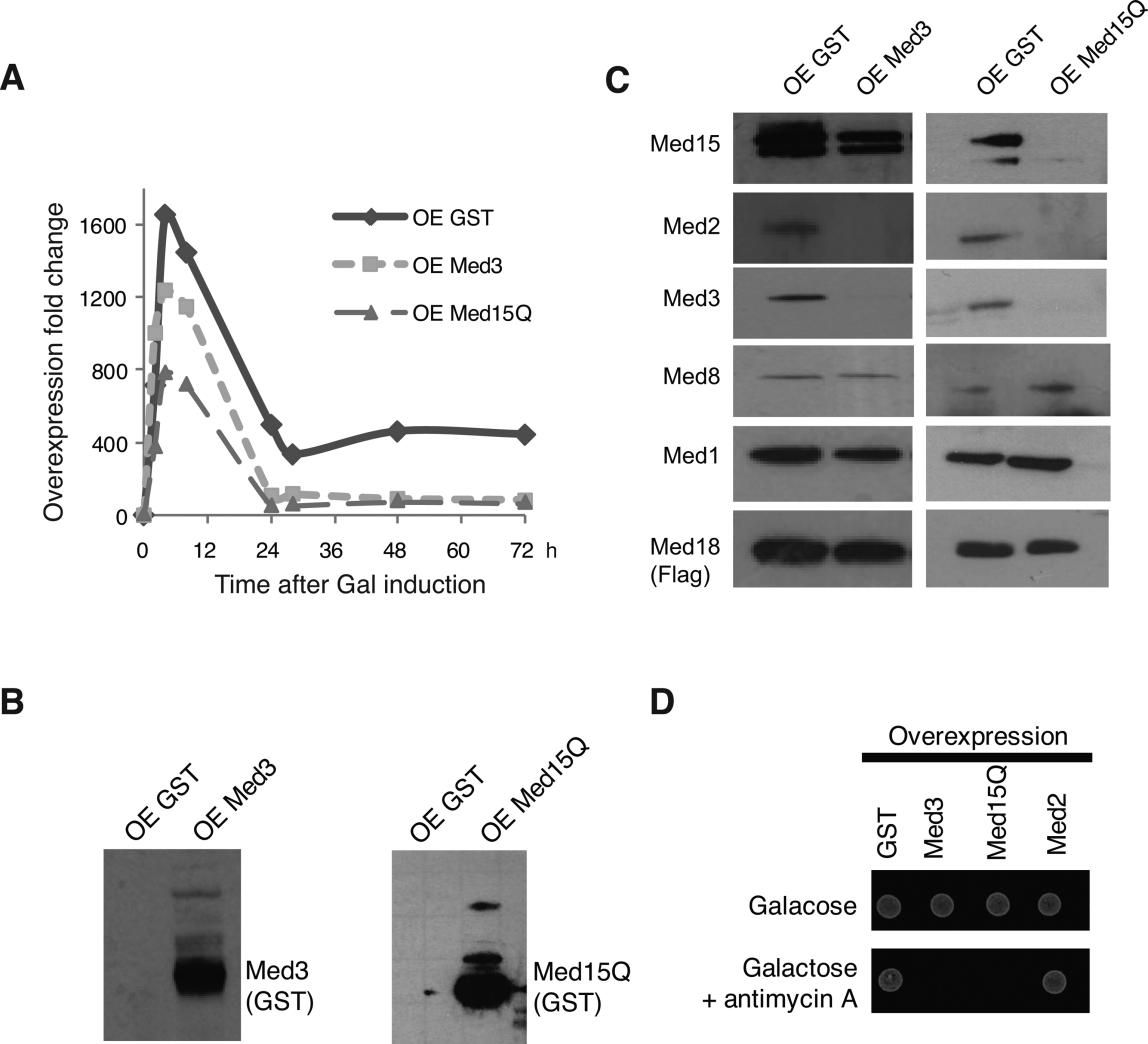
Med3 or Med15Q overexpression destabilizes the Med15 module and impair *GAL* gene induction. (**A**) Quantification by qPCR of galactose induced GST, Med3-GST and MED15Q-GST transcripts. Yeast cells were grown in SGal-ura medium and collected at different time points as indicated. Fold changes were calculated based on the signal in non-induced cells. Actin was used as the loading control. (**B**) GST, Med3-GST and Med15Q-GST proteins were detected by immunoblotting as aggregates in the pellet. (**C**) Immunoblot analysis of Mediator components. Cell lysates were produced from cells overexpressing GST, Med3-GST or Med15Q-GST. The yeast strain used had a Flag-tag on the chromosomal copy of Med18p and Mediator was purified over Flag-M2 agarose (Sigma). Immunoblotting of Flag-Med18p was used as an internal loading control. (**D**) Cells overexpressing Med3-GST or Med15Q-GST failed to grow on galactose medium containing the respiration inhibitor antimycin A.

The Med15 module is required for Gal4 function and galactose induction in yeast (30). In the plasmid constructs used for the overexpression experiments, we had placed the Med3 and Med15Q constructs under the control of the *GAL1*/*0* gene promoter, which in turn was induced by galactose. We therefore expected that our experimental setup could lead to the formation of negative feedback loop. Overexpression of Med3 or Med15Q would cause amyloid formation, leading to loss of the Med15 module from Mediator, which in turn would impair *GAL1/10* promoter activation. The expression patterns of the GST-Med3 and GST-Med15Q constructs supported this idea (Figure 3A). In wt cells, galacose induction of GST expression lead to a biphasic pattern, with a peak of transcript levels after about 5 h (about 1600-fold induction), which then dropped to lower, steady state levels after 24 h (about 400-fold induction). The observed drop is consistent with previous studies of galactose-induced genes (31,32). When we instead monitored GST-Med3 or GST-Med15Q overexpression, the maximum peak of gene transcription was lower and the activated steady state levels of gene transcription about 10% of the levels observed upon GST overexpression.

We next investigated if the amyloid formation observed for overexpressed Med3 and Med15Q could influence the composition of the endogenous Mediator complex. To this end, we purified Mediator from a Flag-Med18 strain in which we had overexpressed Med3-GST or Med15Q-GST. As a negative control, we also overexpressed GST. After cell lysis, Med3 and Med15Q were predominantly detected in the insoluble pellet (Figure 3B). In the supernatant fraction, we could detect Mediator subunits from both the head and middle module (Med1, Med8, Med18), suggesting that these regions of Mediator stay intact. When Med15Q was overexpressed, the entire Med15 module, containing Med2, Med3 and Med15, were lost from Mediator. Med3 overexpression also led to a change in the subunit composition of Mediator, with a loss of Med2 and Med3. Interestingly, Med3 overexpression did not cause a loss of Med15 (Figure 3C). There is thus a clear difference between the effects Med3 and Med15Q overexpression on the subunits composition. However, this differential effect is in perfect agreement with the suggested subunit composition of the Med15 module, in which a heterodimer of Med2 and Med3 is linked to the rest of Mediator via the Med15 protein (12).

The Med15 module is essential for normal *GAL* gene induction and required for growth on galactose in the presence of the respiration inhibitor antimycin A (30). In agreement with the change in Mediator subunit composition upon Med3 or Med15Q overexpression, these cells were also unable to grow on galactose in the presence of antimycin A, demonstrating that endogenous Med3 and Med15 had lost their biological function (Figure 3D). Overexpression of Med2, which also contains a poly-Q domain, did not cause a similar phenotype.

**Figure 4.**
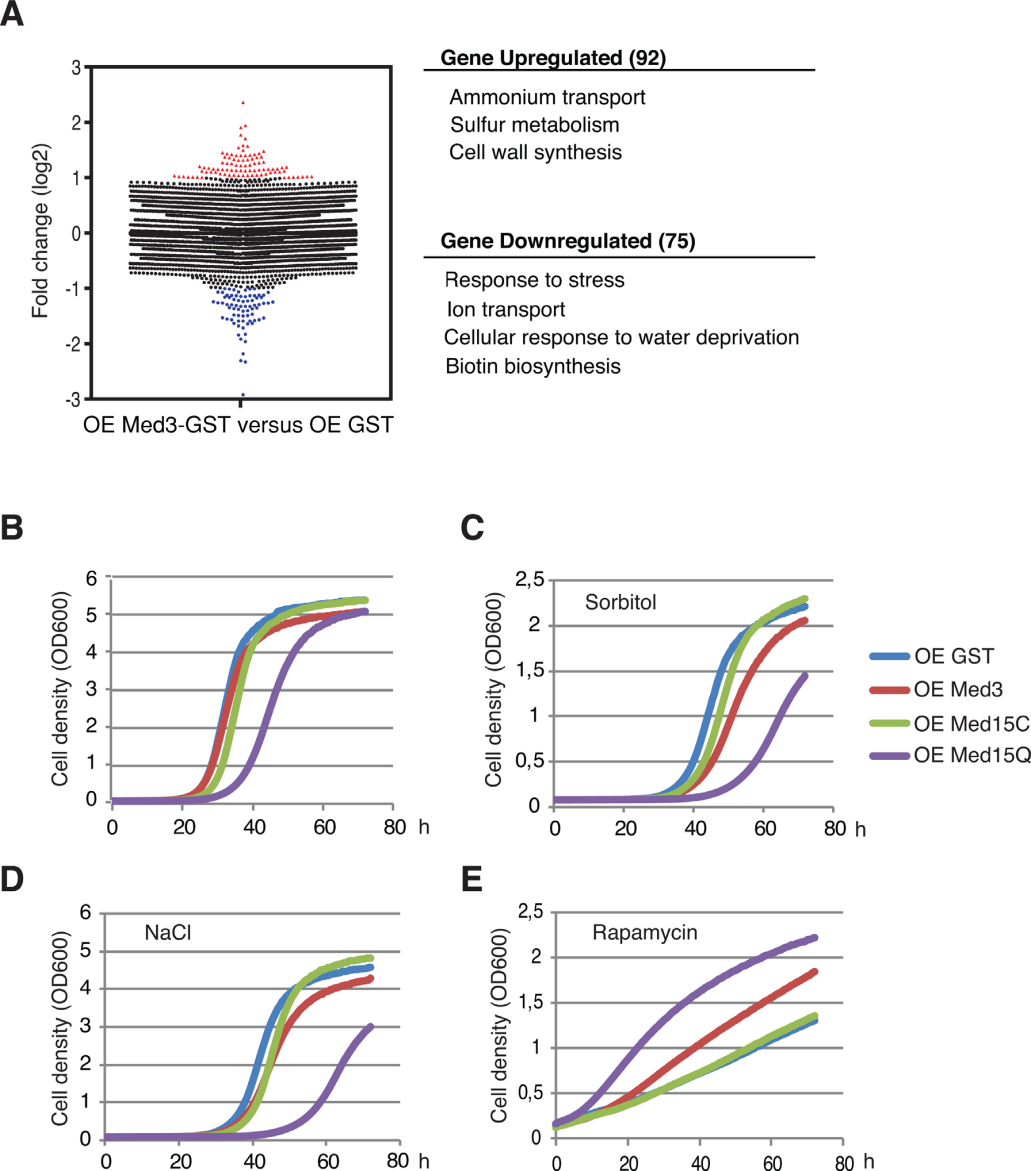
Overexpression of Med3 and Med15Q alter the tolerance to stress conditions. (**A**) Microarray expression profiling of cells overexpressing Med3-GST for 48 h. Fold change (log2 transformed) of gene transcription in Med3-GST versus GST overexpressing cells is presented in a scatter plot. Upregulated and downregulated genes with a 2-fold cutoff are marked by red and blue colors respectively. Gene ontology analysis showed that affected genes could be connected to specific functional categories. *P*-values < 0.001. (**B**) Growth of yeast strains overexpressing (OE) the indicated proteins in SGal-ura medium. (**C**–**E**) Growth of yeast strains overexpressing the indicated proteins in SGal-ura medium supplemented with sorbitol (1.5 M), NaCl (0.5 M) or Rapamycin (10 uM). OD_600_ values were measured every 30 min. The experiments were repeated three times and the presented curves were plotted using the average values.

We next analyzed if amyloid formation affects genome wide transcription patterns using affymetrix microarray analysis. We found that *MED3* overexpression and the accompanying amyloid formation caused a change in the transcript levels of a subset of yeast genes (92 genes upregulated, 75 downregulated) (Figure 4A, Supplementary Tables S3 and S4). Gene ontology analysis with the Princeton GO Term Finder indicated that the downregulated genes were involved in diverse processes, such as biotin synthesis, ion transport, cellular response to water depravation and response to stress (Figure 4A and Supplementary Table S5). An earlier study demonstrated that deletion of *MED3* or *MED15* causes a decrease in osmotic stress resistance (33) and we decided to investigate if overexpression of Med3 or Med15Q caused a similar phenotype by examining growth under osmotic stress (Figure 4B–D). As negative controls, we expressed GST and a C-terminal Med15 fragment, which do not contain the Poly-Q domain (termed Med15C). Overexpression of Med15Q caused a slow growing phenotype, not observed in Med3 overexpressing cells, which is in agreement with the previously reported phenotypes of *med3Δ* and *med15Δ* cells (34). The slow growth phenotype of Med15Q was further accentuated in the presence of Sorbitol and NaCl (osmotic stress). Under these conditions also Med3 overexpressing cells grew slightly slower than the controls. We also investigated effects of antibiotic stress. After addition of rapamycin, Med15 is rapidly recruited to the promoters of rapamycin-induced genes (35). In agreement with this observation, overexpression of *MED3* or *MED15Q* causes increased resistance to rapamycin stress (Figure 4E). The sensitivity to other antibiotics—hygromycin B, kanamycin and nourseothricin—remained unchanged (data not shown).

To ensure that the observed effects were specific and not caused by overexpression of any given poly-Q domain, we also monitored Htt25Q and Htt103Q overexpression under osmotic and rapamycin stress conditions (Supplementary Figure S3). Overexpression of Htt25Q did not affect resistance to osmotic stress or rapamycin. Htt103Q overexpression caused a slow growing phenotype under all conditions tested. The observed effects of Med3 or Med15Q overexpression were thus distinct from those caused by overexpression of the unrelated Htt25Q and Htt103Q proteins.

## DISCUSSION

Mediator was first described as a bridge between activators and the basal transcription machinery present at the promoter. A number of activator-binding domains have been described in Mediator and the complex can be recruited to promoters by gene-specific transcription factors. How Mediator stimulates transcription activity at promoters is still subjected to intensive studies (8). Recruitment of the basal transcription machinery is one important aspect, but Mediator also stimulates other steps in gene expression, including chromatin organization at genes (36,37) and post-translational events such as splicing (38). We have reported that Mediator interacts with nucleosomes *in vitro* and chromatin *in vivo* (19). These conclusions are further supported by our findings here that Mediator interacts with chromatin *in situ*.

The Mediator Med15 module interacts with a number of different activator proteins, including Gcn4 and Gal4 in yeast and Oaf1 in mammalian cells (39). Hence loss of components of the Med15 module may change the interaction with a number of gene-specific transcription factors and influence transcriptional response. A recent study pointed to an interesting and unexpected mechanism involving Med3 and Cdk8, a cyclin dependent kinase found in Mediator. Under specific conditions, Cdk8 can phosphorylate Med3, which in turn triggers Med3 degradation by the Grr1 ubiquitin ligase. This mechanism suppresses Mediator function and thus prevents transcription activation (40). Here we report on an alternative mechanism, which changes the stability of the Med15 module and affects Mediator's ability to support activated transcription. We find that two components of the Mediator Med15 module, Med3 and Med15, can convert to amyloid-like aggregates under oxidative stress. Amyloid formation may also be induced by overexpression of Med3 or a poly-Q rich stretch derived from Med15. Amyloid formation leads to the concomitant loss of Med2/Med3 (in the case of Med3 overexpression) or Med2/3/15 (in the case of Med15Q overexpression). Amyloid formation may thus be an effective way of regulating the presence of the Med15 module, which is an important interaction hub for transcription activators in the Mediator complex.

The idea that amyloid formation may influence regulated gene expression is not without precedence. Sup35 is a translation termination factor (41) and previous studies have demonstrated that aggregate conversion prevents it from associating with ribosomes, which in turn causes translational read-through of stop codons. This effect may lead to higher genetic variability, which can be beneficial under cellular stress conditions (42). Our finding that Med3 and Med15 amyloid formation may influence the subunit composition of the Mediator complex adds another layer of complexity to the role of amyloid formation in regulated gene expression. Amyloid formation and loss of the Med15 module suggests a new epigenetic mechanism for regulation of gene transcription. Yeast cells may exploit protein aggregation mechanisms to alter the transcription output and further increase the survival rate under different stress conditions. Interestingly, Med15's ability to aggregate may be evolutionary conserved. Mutations in the Ataxin-1 encoding gene (*ATXN1*) cause spinocerebellar ataxia type 1, a neurodegenerative disease. The pathogenic form of Ataxin-1 contains an elongated poly-Q stretch that causes spontaneous protein aggregation. Med15 interacts directly with Ataxin-1 and influences poly-Q-induced misfolding and proteotoxicity in a cell model system (43).

## SUPPLEMENTARY DATA

Supplementary Data are available at NAR Online.

SUPPLEMENTARY DATA
