# Supplementary material for: Mediator tail subunits can form amyloid-like aggregates in vivo and affect stress response in yeast
Source: Nucleic Acids Res. 2015 Jul 2;43(15):7306–14. doi: 10.1093/nar/gkv629 (PMC4551914; doi:10.1093/nar/gkv629)
Supplement: SUPPLEMENTARY DATA [file supp_43_15_7306__index.html]

Mediator tail subunits can form amyloid-like aggregates in vivo and affect stress response in yeast — Mediator tail subunits can form amyloid-like aggregates in vivo and affect stress response in yeast — SUPPLEMENTARY DATA 

# Mediator tail subunits can form amyloid-like aggregates *in vivo* and affect stress response in yeast

## SUPPLEMENTARY DATA

- SUPPLEMENTARY DATA
