# Supplementary material for: Mediator tail subunits can form amyloid-like aggregates in vivo and affect stress response in yeast
Source: Nucleic Acids Res. 2015 Jul 2;43(15):7306–14. doi: 10.1093/nar/gkv629 (PMC4551914; doi:10.1093/nar/gkv629)
Supplement: SUPPLEMENTARY DATA [file supp_gkv629_nar-03700-v-2014-File006.pdf]

SUPPLEMENTARY FIGURE LEGENDS

Figure S1. Mediator subunits under normal and H_2O_2 stress condition. GFP fusion proteins were created by homologous recombination and expressed using endogenous promoters. Representative images of single cells demonstrate overlap between GFP signals and nuclear DNA (DAPI). The cells expressing GFP-fused Mediator subunits were also exposed to H_2O_2 (0.03%) for 3 hours, Scale bars = 2 μm.

Figure S2. Overexpression of Med3 and truncated versions of Med15. A. Amino acid sequence of Med3. The C-terminal Poly-Q domain, which was removed from truncated Med3, is boxed. B. Amino acid sequence of Med15. The C terminal Poly-Q domain is boxed. The region of the protein marked in yellow was expressed as the Med15C control. C. Western blots showing the protein levels of full length and truncated Med3-GFP. PGK1 protein was used as the loading control and the average fold expression (+/- SD) relative the loading control is indicated. D. Immunoblotting to verify overexpression of GST, Med3-GST, Med15Q-GST, and Med15C-GST after transfection in budding yeast. Anti-GST antibodies were used to detect the fused proteins.

Figure S3. Overexpression of PolyQ domain proteins in stress conditions.

A. Growth of yeast strains overexpressing (OE) the indicated proteins (GST vector, Htt25Q, and Htt103Q) in SGal-ura medium. B to D. Growth of yeast strains overexpressing the indicated proteins in SGal-ura medium supplemented with sorbitol (1.5 M), NaCl (0.5 M), or Rapamycin (10 μM). OD_{600} values were measured every 30 minutes. The experiments were repeated three times and the presented curves were plotted using the average values. E. The growth of cells overexpressing GST, Htt25Q, or Htt103Q on galactose medium containing the respiration inhibitor antimycin A(1 ug/ml).

SUPPLEMENTARY TABLE LEGENDS

Table S1. Strains used in this study.

Table S2. Stress conditions tested for effects on the nuclear localization of Med3-GFP expressed from its endogenous promoter.

Table S3. Genes upregulated in cells overexpressing *MED3*.

Table S4. Genes downregulated in cells overexpressing *MED3*.

Table S5. Gene ontology analysis of genes downregulated in MED3 overexpressing cells.

Figure S1

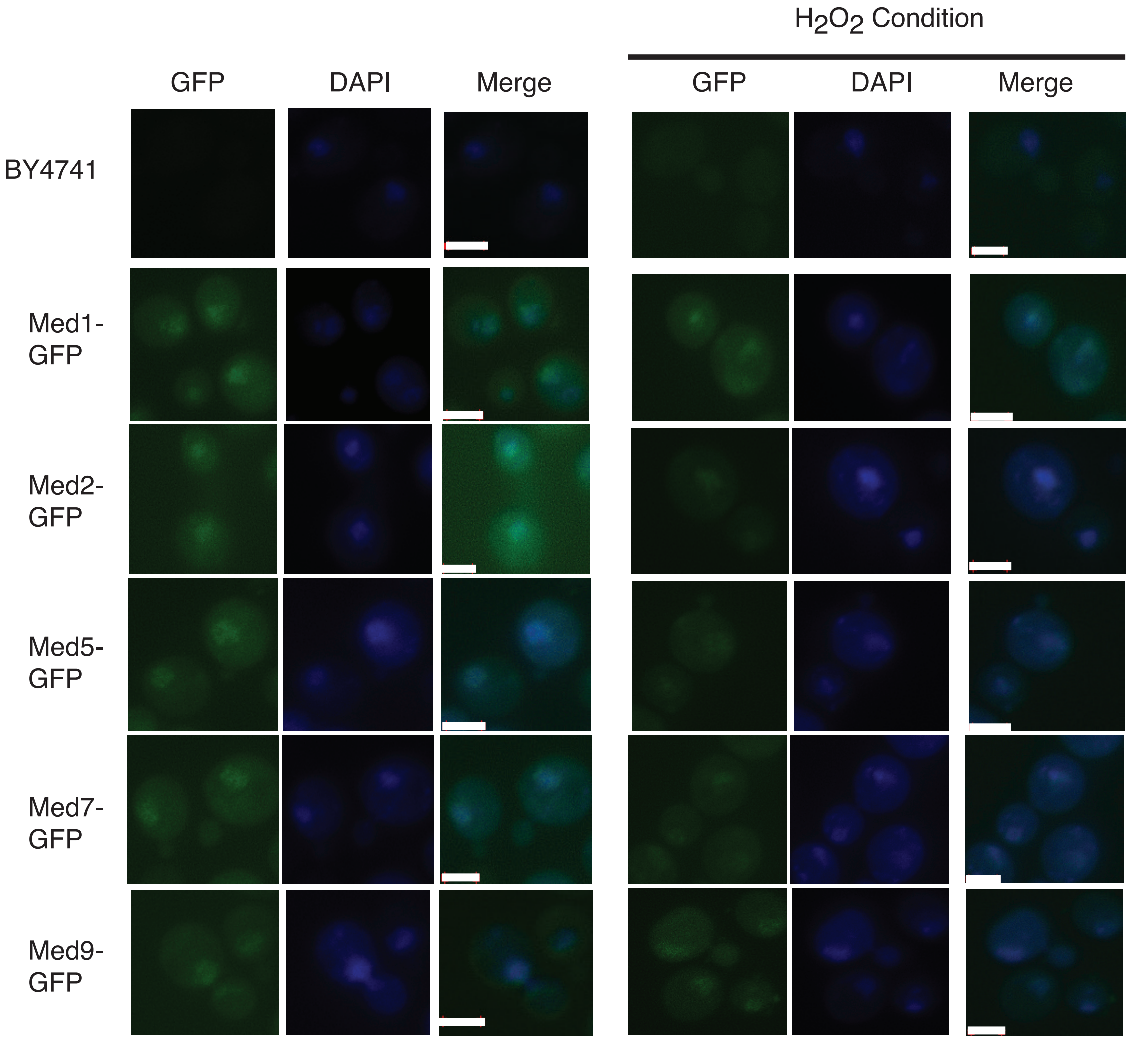

H2O2 Condition
GFP
DAPI
Merge
GFP
DAPI
Merge
BY4741
Med1-GFP
Med2-GFP
Med5-GFP
Med7-GFP
Med9-GFP

Figure S2

A Med3/YGL025C

MDSIIPAGVKLDDLQVILAKNENETRDKVCKQINEARDEILPLRLQFNEFIQIMANIDQE
GSKQADRMAKYLHIRDKILQLNDRFQTLSSHLEALQPLFSTVPEYLKTADNRDRSFQLLE
PLSTYNKNGNAVCSTATVVSTNHSAAASTPTTTATPHANPITHAHSLSNPNSTATMQHNP
LAGKRGPKSGSTMGTPTVHNSTAAAPIAAPKKPRKPRQTKKAKAQAQAQAQAQAQVYAQQ
STVQTPITASMAAALPNPTPSMINSVSPTNVMGTPLTNMMSPMGNAYSMGAQNQGGQVSMSQFNG
SGNGSNPNTNTNSNNTPLQSQLNLNNLTPANILNMSMNNDFQQQQQQQQQQQQPQ
PQYNMNMGMNNMNNGGKELDSLDLNNLELGGLNMDFL

B Med15/YOL051W

MSAAPVQDKDTLSNAERAKNVNGLLQVLMDINTLNGGSSDTADKIRIHAKNFEAALFAKS
SSKKEYMDSMNEKVAVMRNTYNTRKNAVTAAAANNNIKPVEQHHINNLKNSGNSANNMNV
NMNLNPQMFLNQQAQARQQVAQQLRNQQQQQQQQQQQQRRQLTPQQQQLVNQMKVAPIPK
QLLQRIPNIPPNINTWQQVTALAQQKLLTPQDMEAAKEVYKIHQQLLFKARLQQQQAQAQ
AQANNNNNGLPQNGNINNNINIPQQQQMQPPNSSANNNPLQQQSSQNTVPNVLNQINQIF
SPEEQRSLLQEAIETCKNFEKTQLGSTMTEPVKQSFIRKYINQKALRKIQALRDVKNNNN
ANNNGSNLQRAQNVPMNIIQQQQQQNTNNNDTIATSATPNAAAFSQQQNASSKLYQMQQQ
QQAQAQAQAQAQAQAQAQAQAAQAAQAQAQAQAQAQAQAQAQAQAQAQAQAQAQAQAQ
AHAQHQPSQQPQQAQQQPNPLHGLTPTAKDVEVIKQLSLDASKTNLRLTDVTNSLSNEEK
EKIKMKLKQGQKLFVQVSNFAPQVYIITKNENFLKEVFQLRIFVKEILEKCAEGIFVVKL
DTVDRLIIKYQKYWESMRIQILRRQAILRQQQQMANNNGNPGTTSTGNNNNIATQQNMQQ
SLQQMQHLQQLKMQQQQQQQQQQQQQQQQQQQQQQQHIYPSSTPGVANYSAMANAPGNNI
PYMNHKNTSSMDFLNSMENTPKVPVSAAATPSLNKTINGKVNGRTKSNTIPVTSIPSTNK
KLSISNAASQQPTPRSASNTAKSTPNTNPSPLKTQTKNGTPNPNNMKTVQSPMGAQPSYN
SAIIENAFRKEELLLKDLEIRKLEISSRFKHRQEIFKDSPMDLFMSTLGDCLGIKDEEML
TSCTIPKAVVDHINGSGKRKPTKAAQRARDQDSIDISIKDNKLVMKSKFNKSNRSYSIAL
SNVAAIFKGIGGNFKDLSTLVHSSSPSTSSNMDVGNPRKRKASVLEISPQDSIASVLSPD
SNIMSDSKKIKVDSPDDPFMTKSGATTSEKQEVTNEAPFLTSGTSSEQFNVWDWNNWTSA T

C Full length Med3 Tuncated Med3

GFP

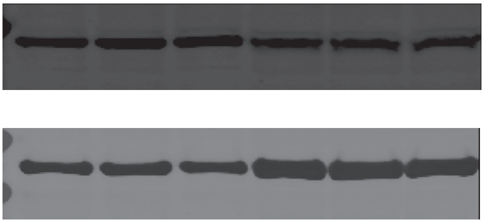

Pgk1

Fold 0.95±0.15 0.48±0.02

D

75KD-

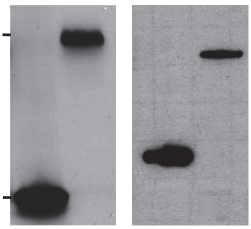

25KD-

GST Med3-GST Med15Q-GST Med15C-GST

Supplementary Figure S3

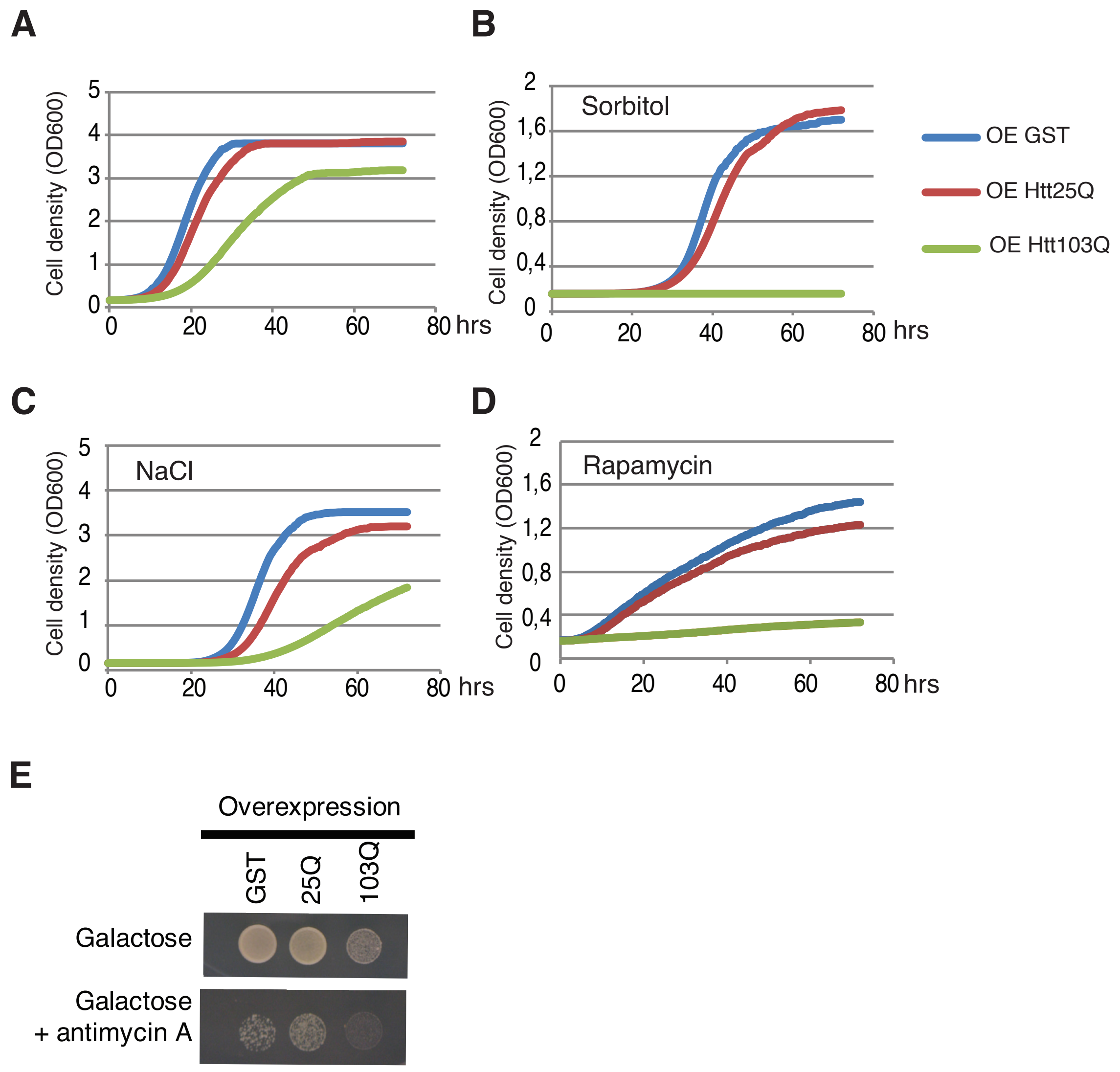

Table S1 Strains used in this study

Name	Genotype	Source
BY4741	MATa; his3Δ 1; leu2Δ 0; met15Δ 0; ura3Δ 0	Euroscarf
sc-29	BY4741,Med18::Med18-3FLAG-KanMX	This study
sc-04	BY4741,Med16::Med16-GFP-HisMX6	Invitrogen
sc-05	BY4741,Med3::Med3-GFP-HisMX6	Invitrogen
sc-06	BY4741,Med15::Med15-GFP-HisMX6	Invitrogen
sc-13	BY4741,Med3::Med3(Truncated)-GFP-NATMX	Invitrogen
sc-22	BY4741,Med2::Med2-GFP-HisMX6	Invitrogen
sc-23	BY4741,Med5::Med5-GFP-HisMX6	Invitrogen
sc-24	BY4741,cycC::cycC-GFP-HisMX6	Invitrogen
sc-27	BY4741,Med1::Med1-GFP-HisMX6	Invitrogen
sc-36	BY4741,Med17::Med17-GFP-HisMX6	Invitrogen
sc-37	BY4741,Med7::Med7-GFP-HisMX6	Invitrogen
sc-38	BY4741,Med8::Med8-GFP-HisMX6	Invitrogen
sc-39	BY4741,Med9::Med9-GFP-HisMX6	Invitrogen
sc-43	BY4741,Skn7::Skn7-GFP-HisMX6	Invitrogen
sc-62	BY4741,pYES2-mHtt103Q-mRFP	reference
sc-49	sc-29, pEGH-GST	This study
sc-50	sc-29, pEGH-Med3-GST	This study
sc-51	sc-29, pEGH-Med15Q-GST	This study
sc-12	sc-05, pEGH-Med3-GST	This study
sc-57	sc-06, pEGH-Med15Q-GST	This study
sc-58	sc-29, pEGH-Med15C-GST	This study
sc-11	sc-05, pEGH-GST	This study

Table S2. Conditions tested for effects on the subcellular localization of Med3-GFP.

Condition	Presumed biological effect(s)	Conc.
Acetic acid	pH stress	50 µM
Acid	pH stress	pH 5.0
Alpha factor	Mating pheromone	10 µM
Base (NaOH)	pH stress	pH 9.0
Caffeine	Purine analog	2 mM
Cobalt chloride	Heavy metal	1.0 mM
$CuSO_4$	Heavy metal	2.0 mg/mL
Deoxycholate	Membrane stress	1 mM
Ethanol	Alcohol stress	5%
Ethanolamine	Reactive amine	2 mM
Ferric chloride	Heavy metal	2.5 mM
Guanidine HCl	Hsp104 inhibition	5 mM
H_2O_2	Oxidative stress	0.03%
Hydroxyurea	Replication stress	25 mM
Hygromycin	Antifungal	0.5 µM
Ibuprofen sodium salt	Non-specific cyclooxygenase inhibition	50 µM
Lithium chloride	Osmotic stress	200 mM
Magnesium chloride	Osmotic stress	150 mM
MG132	Proteasome inhibition	50 µM
Nickel chloride	Heavy metal	2.5 mM
Nicotinic acid	Metabolite	5 mM
Sodium chloride	Osmotic stress	0.5 M
Sodium orthovanadate	Orthophosphate competitor	1 mM
YP Glycerol	Alternative carbon source	2%
YP Maltose	Alternative carbon source	2%
YP Raffinose	Alternative carbon source	2%
Zinc chloride	Diverse mechanisms; interference with metal homeostasis	5 mM

TABLE S3. Genes upregulated by MED3 overexpression.

Probe Set ID:	Standard Name:	Gene product	Fold change	Systematic Name:
1779425_at	CYC3	Cytochrome c heme lyase (holocytochrome c synthase), attaches heme to apo-cytochrome c (Cyc1p or Cyc7p) in the mitochondrial intermembrane space; human ortholog may have a role in microphthalmia with linear skin defects (MLS)	2,065 up	YAL039C
1776338_at	SYN8	Endosomal SNARE related to mammalian syntaxin 8	2,029 up	YAL014C
1773888_at	CYS3	Cystathionine gamma-lyase, catalyzes one of the two reactions involved in the transsulfuration pathway that yields cysteine from homocysteine with the intermediary formation of cystathionine	2,207 up	YAL012W
1777445_at			3,415 up	YAR068W, YHR214W-A
1772654_at	ALG3	Dolichol-P-Man dependent alpha(1-3) mannosyltransferase, involved in the synthesis of dolichol-linked oligosaccharide donor for N-linked glycosylation of proteins	2,484 up	YBL082C, YBL083C
1772126_at	FIG1	Integral membrane protein required for efficient mating; may participate in or regulate the low affinity Ca2+ influx system, which affects intracellular signaling and cell-cell fusion during mating	3,840 up	YBR040W
1772558_at	TIP1	Major cell wall mannoprotein with possible lipase activity; transcription is induced by heat- and cold-shock; member of the Srp1p/Tip1p family of serine-alanine-rich proteins	2,999 up	YBR067C
1779265_at			2,227 up	YBR071W
1779615_at	PHO3	Constitutively expressed acid phosphatase similar to Pho5p; brought to the cell surface by transport vesicles; hydrolyzes thiamin phosphates in the periplasmic space, increasing cellular thiamin uptake; expression is repressed by thiamin	2,538 up	YBR092C
1770074_at			2,146 up	YBR141C
1769377_at			2,187 up	YBR238C
1774721_at	ENP1	Protein associated with U3 and U14 snoRNAs, required for pre-rRNA processing and 40S ribosomal subunit synthesis; localized in the nucleus and concentrated in the nucleolus	2,485 up	YBR247C
1777871_at	IMG1	Mitochondrial ribosomal protein of the large subunit, required for respiration and for maintenance of the mitochondrial genome	2,030 up	YCR046C
1774930_at	THR4	Threonine synthase, conserved protein that catalyzes formation of threonine from O-phosphohomoserine; expression is regulated by the GCN4-mediated general amino acid control pathway	2,233 up	YCR053W
1779136_at	RSA4	WD-repeat protein involved in ribosome biogenesis; may interact with ribosomes; required for maturation and efficient intra-nuclear transport or pre-60S ribosomal subunits, localizes to the nucleolus	2,641 up	YCR072C
1775158_at			2,672 up	YDL241W
1773886_at	PCL2	Cyclin, interacts with cyclin-dependent kinase Pho85p; member of the Pcl1,2-like subfamily, involved in the regulation of polarized growth and morphogenesis and progression through the cell cycle; localizes to sites of polarized cell growth	2,595 up	YDL127W
1778149_at			2,328 up	YDR034W-B
1779112_at	HEM13	Coproporphyrinogen III oxidase, an oxygen requiring enzyme that catalyzes the sixth step in the heme biosynthetic pathway; transcription is repressed by oxygen and heme (via Rox1p and Hap1p)	2,819 up	YDR044W
1776314_at			2,067 up	YDR124W
1771565_at	ATC1	Nuclear protein, possibly involved in regulation of cation stress responses and/or in the establishment of bipolar budding pattern	2,226 up	YDR184C
1778111_at	ATO3	Plasma membrane protein, regulation pattern suggests a possible role in export of ammonia from the cell; phosphorylated in mitochondria; member of the TC 9.B.33 YaaH family of putative transporters	2,188 up	YDR384C
1778685_at	UTP6	Nucleolar protein, component of the small subunit (SSU) processome containing the U3 snoRNA that is involved in processing of pre-18S rRNA	2,214 up	YDR449C
1777030_at			2,190 up	YDR476C
1779506_at	UTR2	Chitin transglycosylase that functions in the transfer of chitin to beta(1-6) and beta(1-3) glucans in the cell wall; similar to and functionally redundant with Crh1; glycosylphosphatidylinositol (GPI)-anchored protein localized to bud neck	2,179 up	YEL040W
1774550_at	ISC1	Mitochondrial membrane localized inositol phosphosphingolipid phospholipase C, hydrolyzes complex sphingolipids to produce ceramide; activated by phosphatidylserine, cardiolipin, and phosphatidylglycerol; mediates Na+ and Li+ halotolerance	2,013 up	YER019W
1776603_at	ILV1	Threonine deaminase, catalyzes the first step in isoleucine biosynthesis; expression is under general amino acid control; ILV1 locus exhibits highly positioned nucleosomes whose organization is independent of known ILV1 regulation	2,046 up	YER086W
1777498_at	SCS2	Integral ER membrane protein that regulates phospholipid metabolism via an interaction with the FFAT motif of Opi1p, also involved in telomeric silencing, disruption causes inositol auxotrophy above 34 degrees C, VAP homolog	2,144 up	YER120W
1775737_at			2,397 up	YER156C
1779410_at	PUG1	Plasma membrane protein with roles in the uptake of protoprophyrin IX and the efflux of heme; expression is induced under both low-heme and low-oxygen conditions; member of the fungal lipid-translocating exporter (LTE) family of proteins	2,149 up	YER185W
1769374_at	MOB2	Component of the RAM signaling network, that activates the Cbk1p kinase, thereby facilitating the Ace2p-dependent daughter cell-specific transcription of genes involved in cell separation; similar to Mob1p	2,598 up	YFL034C-B
1776471_at	MIG2	Protein containing zinc fingers, involved in repression, along with Mig1p, of SUC2 (invertase) expression by high levels of glucose; binds to Mig1p-binding sites in SUC2 promoter	2,736 up	YGL209W
1779094_at	PGD1	Subunit of the RNA polymerase II mediator complex; associates with core polymerase subunits to form the RNA polymerase II holoenzyme; essential for basal and activated transcription; direct target of Cyc8p-Tup1p transcriptional corepressor	16,656 up	YGL025C
1778735_at			2,520 up	YGR035C

1773145_s_at			2,565 up	YGR109W-A, YGR109W-B, YIL080W, YIL082W, YIL082W-A
1771363_at	RTA1	Protein involved in 7-aminocholesterol resistance; has seven potential membrane-spanning regions; expression is induced under both low-heme and low-oxygen conditions; member of the fungal lipid-translocating exporter (LTE) family of protein	2,197 up	YGR213C
1773176_at	DIE2	Dolichyl-phosphoglucose-dependent alpha-1,2 glucosyltransferase of the ER, functions in the pathway that synthesizes the dolichol-linked oligosaccharide precursor for N-linked protein glycosylation, has a role in regulation of ITR1 and INO1	2,269 up	YGR227W
1773748_at	MUP3	Low affinity methionine permease, similar to Mup1p	2,216 up	YHL036W
1778128_at	DUR3	Plasma membrane transporter for both urea and polyamines, expression is highly sensitive to nitrogen catabolite repression and induced by allophanate, the last intermediate of the allantoin degradative pathway	2,052 up	YHL016C
1777020_at	CIC1	Essential protein that interacts with proteasome components and has a potential role in proteasome substrate specificity; also copurifies with 66S pre-ribosomal particles	2,019 up	YHR052W
1769730_at	FUR1	Uracil phosphoribosyltransferase, synthesizes UMP from uracil; involved in the pyrimidine salvage pathway	2,415 up	YHR128W
1777918_at	POG1	Putative transcriptional activator that promotes recovery from pheromone induced arrest; inhibits both alpha-factor induced G1 arrest and repression of CLN1 and CLN2 via SCB/MCB promoter elements; potential Cdc28p substrate; SBF regulated	2,028 up	YIL122W
1773613_at	OPT1	Proton-coupled oligopeptide transporter of the plasma membrane; also transports glutathione and phytochelatin; member of the OPT family	5,133 up	YJL212C
1779514_at	ALB1	Shuttling pre-60S factor; involved in the biogenesis of ribosomal large subunit; interacts directly with Arx1p; responsible for Tif6p recycling defects in absence of Rei1p	2,392 up	YJL122W
1778954_at	RPE1	D-ribulose-5-phosphate 3-epimerase, catalyzes a reaction in the non-oxidative part of the pentose-phosphate pathway; mutants are sensitive to oxidative stress	2,269 up	YJL121C
1773302_at	ESS1	Peptidylprolyl-cis/trans-isomerase (PPIase) specific for phosphorylated serine and threonine residues N-terminal to proline; regulates phosphorylation of the RNA polymerase II large subunit (Rpo21p) C-terminal domain	2,187 up	YJR017C
1779407_at	ANB1	Translation elongation factor eIF-5A, previously thought to function in translation initiation; similar to and functionally redundant with Hyp2p; undergoes an essential hypusination modification; expressed under anaerobic conditions	2,351 up	YJR047C
1778487_at	RPA12	RNA polymerase I subunit A12.2; contains two zinc binding domains, and the N terminal domain is responsible for anchoring to the RNA pol I complex	2,710 up	YJR063W
1772275_at	JJJ3	Protein of unknown function, contains a J-domain, which is a region with homology to the E. coli DnaJ protein	2,017 up	YJR097W
1778802_at	NNF1	Essential component of the MIND kinetochore complex (Mtw1p Including Nnf1p-Nsl1p-Dsn1p) which joins kinetochore subunits contacting DNA to those contacting microtubules; required for accurate chromosome segregation	2,697 up	YJR112W
1773710_at			2,576 up	YJR116W
1770848_at	DPH2	Protein required, along with Dph1p, Kti11p, Jjj3p, and Dph5p, for synthesis of diphthamide, which is a modified histidine residue of translation elongation factor 2 (Eft1p or Eft2p); may act in a complex with Dph1p and Kti11p	2,010 up	YKL191W
1772556_at	PIR1	O-glycosylated protein required for cell wall stability; attached to the cell wall via beta-1,3-glucan; mediates mitochondrial translocation of Apn1p; expression regulated by the cell integrity pathway and by Swi5p during the cell cycle	2,089 up	YKL164C
1773734_at	RCN1	Protein involved in calcineurin regulation during calcium signaling; has similarity to H. sapiens DSCR1 which is found in the Down Syndrome candidate region	2,250 up	YKL159C
1770480_at	GAP1	General amino acid permease; localization to the plasma membrane is regulated by nitrogen source	2,385 up	YKR039W
1777847_at			2,145 up	YKR045C
1778293_at			2,506 up	YKR075C
1769945_at	SRP40	Nucleolar, serine-rich protein with a role in preribosome assembly or transport; may function as a chaperone of small nucleolar ribonucleoprotein particles (snoRNPs); immunologically and structurally to rat Nopp140	2,071 up	YKR092C
1776767_at	MHT1	S-methylmethionine-homocysteine methyltransferase, functions along with Sam4p in the conversion of S-adenosylmethionine (AdoMet) to methionine to control the methionine/AdoMet ratio	2,343 up	YLL062C
1778233_at	MMP1	High-affinity S-methylmethionine permease, required for utilization of S-methylmethionine as a sulfur source; has similarity to S-adenosylmethionine permease Sam3p	2,640 up	YLL061W
1771069_at			2,910 up	YLR063W
1776414_at	ICT1	Lysophosphatidic acid acyltransferase, responsible for enhanced phospholipid synthesis during organic solvent stress; null displays increased sensitivity to Calcofluor white; highly expressed during organic solvent stress	2,028 up	YLR099C
1777580_at	RNH203	Ribonuclease H2 subunit, required for RNase H2 activity; related to human AGS3 that causes Aicardi-Goutieres syndrome	2,173 up	YLR154C
1772232_at			2,133 up	YLR179C
1778359_at	SAM1	S-adenosylmethionine synthetase, catalyzes transfer of the adenosyl group of ATP to the sulfur atom of methionine; one of two differentially regulated isozymes (Sam1p and Sam2p)	2,786 up	YLR180W
1770954_at			2,053 up	YLR363W-A
1775717_at			2,015 up	YLR413W
1778286_at			2,053 up	YLR466C-B
1778724_x_at			2,075 up	YLR466C-B
1769317_at	DAT1	DNA binding protein that recognizes oligo(dA).oligo(dT) tracts; Arg side chain in its N-terminal pentad Gly-Arg-Lys-Pro-Gly repeat is required for DNA-binding; not essential for viability	2,177 up	YML113W

1780107_at	GIM5	Subunit of the heterohexameric cochaperone prefoldin complex which binds specifically to cytosolic chaperonin and transfers target proteins to it	2,306 up	YML094W
1776837_at	HUG1	Protein involved in the Mec1p-mediated checkpoint pathway that responds to DNA damage or replication arrest, transcription is induced by DNA damage	2,060 up	YML058W-A
1777384_at	PRM6	Pheromone-regulated protein, predicted to have 2 transmembrane segments; regulated by Ste12p during mating	2,678 up	YML047C
1776558_at	HXT2	High-affinity glucose transporter of the major facilitator superfamily, expression is induced by low levels of glucose and repressed by high levels of glucose	10,772 up	YMR011W
1776498_at	UTP15	Nucleolar protein, component of the small subunit (SSU) processome containing the U3 snoRNA that is involved in processing of pre-18S rRNA	2,432 up	YMR093W
1779680_at			2,966 up	YMR310C
1773356_at	MEP2	Ammonium permease involved in regulation of pseudohyphal growth; belongs to a ubiquitous family of cytoplasmic membrane proteins that transport only ammonium (NH4+); expression is under the nitrogen catabolite repression regulation	3,264 up	YNL142W
1771542_at	RPC19	RNA polymerase subunit AC19, common to RNA polymerases I and III	2,642 up	YNL113W
1779113_at	EOS1	Protein involved in N-glycosylation; deletion mutation confers sensitivity to exidative stress and shows synthetic lethality with mutations in the spindle checkpoint genes BUB3 and MAD1; YNL080C is not an essential gene	2,001 up	YNL080C
1774158_at	MLF3	Serine-rich protein of unknown function, predicted to be palmitoylated; overproduction suppresses the growth inhibition caused by exposure to the immunosuppressant leflunomide	2,014 up	YNL074C
1776108_at			2,093 up	YNL046W
1776356_at	BOP3	Protein of unknown function, potential Cdc28p substrate; overproduction confers resistance to methylmercury	2,226 up	YNL042W
1776673_at	NCE103	Carbonic anhydrase; poorly transcribed under aerobic conditions and at an undetectable level under anaerobic conditions; involved in non-classical protein export pathway	2,284 up	YNL036W
1778591_at	AGA1	Anchorage subunit of a-agglutinin of a-cells, highly O-glycosylated protein with N-terminal secretion signal and C-terminal signal for addition of GPI anchor to cell wall, linked to adhesion subunit Aga2p via two disulfide bonds	3,746 up	YNR044W
1775697_at			2,014 up	YNR064C
1779461_at	MDY2	Protein with a role in insertion of tail-anchored proteins into the ER membrane; forms a complex with Get4p; required for efficient mating; involved in shmoo formation and nuclear migration in the pre-zygote; associates with ribosomes	2,324 up	YOL111C
1773669_at	UTP23	Essential nucleolar protein that is a component of the SSU (small subunit) processome involved in 40S ribosomal subunit biogenesis; has homology to PINc domain protein Fcf1p, although the PINc domain of Utp23p is not required for function	2,205 up	YOR004W
1769549_at	FSF1	Putative protein, predicted to be an alpha-isopropylmalate carrier; belongs to the sideroblastic-associated protein family; non-tagged protein is detected in purified mitochondria; likely to play a role in iron homeostasis	2,148 up	YOR271C
1773158_at	VTS1	Post-transcriptional gene regulator, flap-structured DNA-binding and RNA-binding protein; shows genetic interactions with Vti1p, a v-SNARE involved in cis-Golgi membrane traffic; stimulates Dna2p endonuclease activity; contains a SAM domain	2,411 up	YOR359W
1776763_at	CLN2	G1 cyclin involved in regulation of the cell cycle; activates Cdc28p kinase to promote the G1 to S phase transition; late G1 specific expression depends on transcription factor complexes, MBF (Swi6p-Mbp1p) and SBF (Swi6p-Swi4p)	2,101 up	YPL256C
1772608_at	PRM3	Pheromone-regulated protein required for nuclear envelope fusion during karyogamy; localizes to the outer face of the nuclear membrane; interacts with Kar5p at the spindle pole body	2,234 up	YPL192C
1778654_at	CAR1	Arginase, responsible for arginine degradation, expression responds to both induction by arginine and nitrogen catabolite repression; disruption enhances freeze tolerance	2,006 up	YPL111W
1774976_at	NOC4	Nucleolar protein, forms a complex with Nop14p that mediates maturation and nuclear export of 40S ribosomal subunits	2,647 up	YPR144C

TABLE S4. Genes downregulated by MED3 overexpression.

Probe Set ID:	Standard Name:	Gene product	Fold change	Systematic Name:
1776156_at	BDH2	Putative medium-chain alcohol dehydrogenase with similarity to BDH1; transcription induced by constitutively active PDR1 and PDR3	2,987 down	YAL061W
1778729_at			2,013 down	YAR029W
1779568_at	REG2	Regulatory subunit of the Glc7p type-1 protein phosphatase; involved with Reg1p, Glc7p, and Snf1p in regulation of glucose-repressible genes, also involved in glucose-induced proteolysis of maltose permease	2,656 down	YBR050C
1777569_at	HSP26	Small heat shock protein (sHSP) with chaperone activity; forms hollow, sphere-shaped oligomers that suppress unfolded proteins aggregation; oligomer activation requires a heat-induced conformational change; not expressed in unstressed cells	3,894 down	YBR072W
1779985_at	PHO5	Repressible acid phosphatase (1 of 3) that also mediates extracellular nucleotide-derived phosphate hydrolysis; secretory pathway derived cell surface glycoprotein; induced by phosphate starvation and coordinately regulated by PHO4 and PHO2	2,824 down	YBR093C
1779364_at	RTC2	Protein of unknown function; identified in a screen for mutants with decreased levels of rDNA transcription; detected in highly purified mitochondria; null mutant suppresses cdc13-1; similar to a G-protein coupled receptor from S. pombe	2,327 down	YBR147W
1770353_at	GIT1	Plasma membrane permease, mediates uptake of glycerophosphoinositol and glycerophosphocholine as sources of the nutrients inositol and phosphate; expression and transport rate are regulated by phosphate and inositol availability	3,770 down	YCR098C
1779266_at	RTN2	Protein of unknown function; has similarity to mammalian reticulon proteins; member of the RTNLA (reticulon-like A) subfamily	4,944 down	YDL204W
1776528_at	GPM2	Homolog of Gpm1p phosphoglycerate mutase, which converts 3-phosphoglycerate to 2-phosphoglycerate in glycolysis; may be non-functional derivative of a gene duplication event	2,501 down	YDL021W
1779563_at	FMP16	Putative protein of unknown function; proposed to be involved in responding to conditions of stress; the authentic, non-tagged protein is detected in highly purified mitochondria in high-throughput studies	3,098 down	YDR070C
1776550_at	HSP42	Small heat shock protein (sHSP) with chaperone activity; forms barrel-shaped oligomers that suppress unfolded protein aggregation; involved in cytoskeleton reorganization after heat shock	2,552 down	YDR171W
1778969_x_at	PAU2	Member of the seripauperin multigene family encoded mainly in subtelomeric regions, active during alcoholic fermentation, regulated by anaerobiosis, negatively regulated by oxygen, repressed by heme	2,097 down	YEL049W
1780079_at	YEF1	ATP-NADH kinase; phosphorylates both NAD and NADH; homooctameric structure consisting of 60-kDa subunits; sequence similarity to Utr1p and Pos5p; overexpression complements certain pos5 phenotypes	2,447 down	YEL041W
1774137_at	CYC7	Cytochrome c isoform 2, expressed under hypoxic conditions; electron carrier of the mitochondrial intermembrane space that transfers electrons from ubiquinone-cytochrome c oxidoreductase to cytochrome c oxidase during cellular respiration	2,783 down	YEL039C
1779924_at			3,200 down	YER053C-A
1771802_at	GIP2	Putative regulatory subunit of the protein phosphatase Glc7p, involved in glycogen metabolism; contains a conserved motif (GVNK motif) that is also found in Gac1p, Pig1p, and Pig2p	2,217 down	YER054C
1770202_at	SSA4	Heat shock protein that is highly induced upon stress; plays a role in SRP-dependent cotranslational protein-membrane targeting and translocation; member of the HSP70 family; cytoplasmic protein that concentrates in nuclei upon starvation	2,103 down	YER103W
1769579_at	GPG1	Proposed gamma subunit of the heterotrimeric G protein that interacts with the receptor Gpr1p; involved in regulation of pseudohyphal growth; requires Gpb1p or Gpb2p to interact with Gpa2p; overproduction causes prion curing	2,033 down	YGL121C
1774777_at	NQM1	Transaldolase of unknown function; transcription is repressed by Mot1p and induced by alpha-factor and during diauxic shift	3,006 down	YGR043C
1772958_at	FMP48	Putative protein of unknown function; the authentic, non-tagged protein is detected in highly purified mitochondria in high-throughput studies; induced by treatment with 8-methoxypsoralen and UVA irradiation	2,961 down	YGR052W
1774240_at	SPR3	Sporulation-specific homolog of the yeast CDC3/10/11/12 family of bud neck microfilament genes; septin protein involved in sporulation; regulated by ABFI	2,172 down	YGR059W
1775820_at	VHT1	High-affinity plasma membrane H+-biotin (vitamin H) symporter; mutation results in fatty acid auxotrophy; 12 transmembrane domain containing major facilitator subfamily member; mRNA levels negatively regulated by iron deprivation and biotin	2,962 down	YGR065C
1769955_at	CTT1	Cytosolic catalase T, has a role in protection from oxidative damage by hydrogen peroxide	2,038 down	YGR088W
1769723_at			3,011 down	YGR174W-A
1777740_x_at			2,890 down	YGR174W-A
1773961_at	GND2	6-phosphogluconate dehydrogenase (decarboxylating), catalyzes an NADPH regenerating reaction in the pentose phosphate pathway; required for growth on D-glucono-delta-lactone	2,397 down	YGR256W
1776679_at	ARN2	Transporter, member of the ARN family of transporters that specifically recognize siderophore-iron chelates; responsible for uptake of iron bound to the siderophore triacetylfusarinine C	2,355 down	YHL047C
1775842_at	AIM17	Putative protein of unknown function; the authentic, non-tagged protein is detected in highly purified mitochondria in high-throughput studies; null mutant displays reduced frequency of mitochondrial genome loss	2,087 down	YHL021C
1778153_at			2,471 down	YHR022C
1775402_at	SPL2	Protein with similarity to cyclin-dependent kinase inhibitors; downregulates low-affinity phosphate transport during phosphate limitation; overproduction suppresses a plc1 null mutation; GFP-fusion protein localizes to the cytoplasm	2,447 down	YHR136C
1771373_at	SPS100	Protein required for spore wall maturation; expressed during sporulation; may be a component of the spore wall; expression also induced in cells treated with the mycotoxin patulin	2,236 down	YHR139C

1775925_at	OM45	Protein of unknown function, major constituent of the mitochondrial outer membrane; located on the outer (cytosolic) face of the outer membrane	2,641 down	YIL136W
1777218_at	RPI1	Putative transcriptional regulator; overexpression suppresses the heat shock sensitivity of wild-type RAS2 overexpression and also suppresses the cell lysis defect of an mpk1 mutation	2,576 down	YIL119C
1775912_at	COX5B	Subunit Vb of cytochrome c oxidase, which is the terminal member of the mitochondrial inner membrane electron transport chain; predominantly expressed during anaerobic growth while its isoform Va (Cox5Ap) is expressed during aerobic growth	2,502 down	YIL111W
1772391_at	RGI2	Putative protein of unknown function; expression induced under carbon limitation and repressed under high glucose	2,401 down	YIL057C
1777366_at	PIG2	Putative type-1 protein phosphatase targeting subunit that tethers Glc7p type-1 protein phosphatase to Gsy2p glycogen synthase	2,934 down	YIL045W
1778832_at			2,808 down	YIR042C
1771925_at	TPK1	cAMP-dependent protein kinase catalytic subunit; promotes vegetative growth in response to nutrients via the Ras-cAMP signaling pathway; inhibited by regulatory subunit Bcy1p in the absence of cAMP; partially redundant with Tpk2p and Tpk3p	2,002 down	YJL164C
1774435_at	FMP33	Putative protein of unknown function; the authentic, non-tagged protein is detected in highly purified mitochondria in high-throughput studies	2,009 down	YJL161W
1778931_at			2,186 down	YJR096W
1777575_at			2,188 down	YJR115W
1779327_at			2,015 down	YJR154W
1774749_at	AAD10	Putative aryl-alcohol dehydrogenase with similarity to P. chrysosporium aryl-alcohol dehydrogenase; mutational analysis has not yet revealed a physiological role	2,597 down	YJR155W
1776204_at			2,402 down	YKL151C
1770886_at			2,252 down	YLR030W
1779091_at			2,343 down	YLR031W
1778656_at	EMP46	Integral membrane component of endoplasmic reticulum-derived COPII-coated vesicles, which function in ER to Golgi transport	2,189 down	YLR080W
1778375_at	TIS11	mRNA-binding protein expressed during iron starvation; binds to a sequence element in the 3'-untranslated regions of specific mRNAs to mediate their degradation; involved in iron homeostasis	3,277 down	YLR136C
1772447_at	SYM1	Protein required for ethanol metabolism; induced by heat shock and localized to the inner mitochondrial membrane; homologous to mammalian peroxisomal membrane protein Mpv17	2,172 down	YLR251W
1775014_at	MSC1	Protein of unknown function; mutant is defective in directing meiotic recombination events to homologous chromatids; the authentic, non-tagged protein is detected in highly purified mitochondria and is phosphorylated	2,898 down	YML128C
1774756_at	TSL1	Large subunit of trehalose 6-phosphate synthase (Tps1p)/phosphatase (Tps2p) complex, which converts uridine-5'-diphosphoglucose and glucose 6-phosphate to trehalose, homologous to Tps3p and may share function	2,230 down	YML100W
1775890_at			2,581 down	YMR090W
1769805_at	ALD3	Cytoplasmic aldehyde dehydrogenase, involved in beta-alanine synthesis; uses NAD+ as the preferred coenzyme; very similar to Ald2p; expression is induced by stress and repressed by glucose	2,361 down	YMR169C
1774019_at	SIP18	Phospholipid-binding protein; expression is induced by osmotic stress	2,370 down	YMR175W
1774157_at	URA10	Minor orotate phosphoribosyltransferase (OPRTase) isozyme that catalyzes the fifth enzymatic step in the de novo biosynthesis of pyrimidines, converting orotate into orotidine-5'-phosphate; major OPRTase encoded by URA5	2,804 down	YMR271C
1772853_at	FET4	Low-affinity Fe(II) transporter of the plasma membrane	3,010 down	YMR319C
1770073_s_at	HSP32, HSP33, SNO4	Possible chaperone and cysteine protease with similarity to E. coli Hsp31 and S. cerevisiae Hsp31p, Hsp33p, and Sno4p, member of the DJ-1/ThiJ/PfpI superfamily, which includes human DJ-1 involved in Parkinson's disease; Possible chaperone and cysteine protease with similarity to E. coli Hsp31 and S. cerevisiae Hsp31p, Hsp32p, and Sno4p, member of the DJ-1/ThiJ/PfpI superfamily, which includes human DJ-1 involved in Parkinson's disease; Possible chaperone and cysteine protease, similar to bacterial Hsp31 and yeast Hsp31p, Hsp32p, and Hsp33p, DJ-1/ThiJ/PfpI superfamily member, predicted involvement in pyridoxine metabolism, induced by mild heat stress and copper deprivation	2,328 down	YMR322C, YOR391C, YPL280W
1777106_at	GOR1	Glyoxylate reductase; null mutation results in increased biomass after diauxic shift; the authentic, non-tagged protein is detected in highly purified mitochondria in high-throughput studies	2,480 down	YNL274C
1780105_at	YTP1	Probable type-III integral membrane protein of unknown function, has regions of similarity to mitochondrial electron transport proteins	2,545 down	YNL237W
1774614_at			2,173 down	YNL200C
1775810_at			3,581 down	YNL194C
1773966_at	YPT53	Rab family GTPase, similar to Ypt51p and Ypt52p and to mammalian rab5; required for vacuolar protein sorting and endocytosis	2,410 down	YNL093W
1777152_at	BIO5	Putative transmembrane protein involved in the biotin biosynthesis pathway; responsible for uptake of 7-keto 8-aminopelargonic acid; BIO5 is in a cluster of 3 genes (BIO3, BIO4, and BIO5) that mediate biotin synthesis	7,558 down	YNR056C
1772916_at	BIO4	Dethiobiotin synthetase, catalyzes the third step in the biotin biosynthesis pathway; BIO4 is in a cluster of 3 genes (BIO3, BIO4, and BIO5) that mediate biotin synthesis; expression appears to be repressed at low iron levels	4,538 down	YNR057C
1772199_at	BIO3	7,8-diamino-pelargonic acid aminotransferase (DAPA), catalyzes the second step in the biotin biosynthesis pathway; BIO3 is in a cluster of 3 genes (BIO3, BIO4, and BIO5) that mediate biotin synthesis	5,025 down	YNR058W
1772997_at	GRE2	3-methylbutanal reductase and NADPH-dependent methylglyoxal reductase (D-lactaldehyde dehydrogenase); stress induced (osmotic, ionic, oxidative, heat shock and heavy metals); regulated by the HOG pathway	2,452 down	YOL151W
1773889_at			2,186 down	YOL131W

1774983_at	ATG34	Receptor protein involved in selective autophagy during starvation; specifically involved in the transport of cargo protein alpha-mannosidase (Ams1p); Atg19p paralog	2,097 down	YOL083W
1775066_at	DCS2	Non-essential, stress induced regulatory protein containing a HIT (histidine triad) motif; modulates m7G-oligoribonucleotide metabolism; inhibits Dcs1p; regulated by Msn2p, Msn4p, and the Ras-cAMP-cAPK signaling pathway, similar to Dcs1p.	3,112 down	YOR173W
1778930_at			2,240 down	YOR289W
1776583_at	FIT2	Mannoprotein that is incorporated into the cell wall via a glycosylphosphatidylinositol (GPI) anchor, involved in the retention of siderophore-iron in the cell wall	3,552 down	YOR382W
1779057_at	UIP4	Protein that interacts with Ulp1p, a Ubl (ubiquitin-like protein)-specific protease for Smt3p protein conjugates; detected in a phosphorylated state in the mitochondrial outer membrane; also detected in ER and nuclear envelope	3,092 down	YPL186C
1779718_at	RNY1	Vacuolar RNase of the T(2) family, relocalizes to the cytosol where it cleaves tRNAs upon oxidative or stationary phase stress; promotes apoptosis under stress conditions and this function is independent of its catalytic activity	2,298 down	YPL123C
1775734_at	CTR1	High-affinity copper transporter of the plasma membrane, mediates nearly all copper uptake under low copper conditions; transcriptionally induced at low copper levels and degraded at high copper levels	2,762 down	YPR124W
1771645_at	SUE1	Mitochondrial protein required for degradation of unstable forms of cytochrome c	2,502 down	YPR151C
1779850_at	GPH1	Non-essential glycogen phosphorylase required for the mobilization of glycogen, activity is regulated by cyclic AMP-mediated phosphorylation, expression is regulated by stress-response elements and by the HOG MAP kinase pathway	2,207 down	YPR160W

Table S5. Gene ontology analysis of genes downregulated in *MED3* overexpressing cells.

Gene ontology biological process.	p-value	Genes annoted to the process	Cluster frequency
biotin biosynthetic process [GO:0009102]	4.58e-05	BIO5 BIO4 BIO3	3 out of 7
cellular response to water deprivation [GO:0042631]	0.0007327	CTT1 SIP18	2 out of 4
oxidation-reduction process [GO:0055114]	0.0008304	BDH2 CTT1 GND2 AIM17 COX5B YJR096W AAD10 ALD3 GOR1 GRE2	10 out of 272
iron ion homeostasis [GO:0055072]	0.002919	ARN2 FET4 FIT2	3 out of 26
siderophore transport [GO:0015891]	0.003321	ARN2 FIT2	2 out of 8
copper ion import [GO:0015677]	0.003321	FET4 CTR1	2 out of 8
response to stress [GO:0006950]	0.006892	HSP26 HSP42 PAU2 SSA4 CTT1 TSL1	6 out of 152
mitochondrial electron transport [GO:0006123]	0.007605	CYC7 COX5B	2 out of 12
Ras protein signal transduction [GO:0007265]	0.008923	RPI1 TPK1	2 out of 13
